# Selective Adsorption of CH_4_/N_2_ on Ni-based MOF/SBA-15 Composite Materials

**DOI:** 10.3390/nano9020149

**Published:** 2019-01-25

**Authors:** Hong Liu, Wei Ding, Shaohua Lei, Xupei Tian, Fubao Zhou

**Affiliations:** 1 Jiangsu Key Laboratory of Fire Safety in Urban Underground Space, China University of Mining and Technology, Xuzhou 221116, China; dw94cumt@163.com (W.D.); 15150028979@163.com (S.L.); txp@cumt.edu.cn (X.T.); zfbcumt@163.com (F.Z.); 2 National Engineering Research Center for Coal Gas Control, China University of Mining and Technology, Xuzhou 221116, China

**Keywords:** Coalbed methane, selective adsorption, spherical SBA-15, MOF

## Abstract

Spherical SBA-15-based metal–organic framework (MOF) composite materials were prepared, with nickel as the metal center of MOFs. The materials were characterized via scanning electron microscopy, X-ray fluorescence analysis, X-ray powder diffraction, Fourier-transform infrared spectroscopy, and nitrogen (N_2_) adsorption–desorption. The methane (CH_4_) or N_2_ high-pressure adsorption isotherms of the samples were measured and compared. The specific surface area and adsorption capacity of the composite materials were generally higher than the pristine MOFs, but were much lower than the synthesized SBA-15. The selectivity of the samples toward a binary gas mixture was determined from the Langmuir adsorption equation. The results revealed that, of all the samples, the MOF-2/SBA-15 sample had the best CH_4_/N_2_ adsorption selectivity, with an adsorption selection parameter (*S*) of 11.1. However, the adsorption of MOF-2/SBA-15 was less than that of spherical SBA-15, due to partial plugging of the pores during the synthesis process. Further research is essential for improving the performance of spherical SBA-15-based MOF materials and (in turn) the enrichment of CH_4_ from the CH_4_/N_2_ mixture.

## 1. Introduction

Interest in clean energy research has increased owing to the growing threat of global warming resulting from the harmful emissions of greenhouse gases. The main ingredient of natural gas, methane (CH_4_), is considered an important substitute to petroleum [1]. Coalbed methane (CBM), a type of natural gas adsorbed onto coalbed with huge reserves, consists mainly of methane and nitrogen (N_2_), and also contains minor components such as carbon dioxide (CO_2_) and water [2]. 

The concentration of methane plays a vital role in the selection of the CBM approach utilized [3], and an extremely low concentration would limit this utilization. For example, ventilation air methane is usually released directly into the atmosphere, as technology for its use is lacking, which results in a serious greenhouse effect [4]. High-efficiency enrichment and application of methane from low-concentration CBM is essential for remitting the worrisome energy crisis, and could reduce greenhouse gas emissions, with great comprehensive benefits for energy, environmental protection, and society. In order to meet the quality required for civil use, the concentration of methane in CBM needs to be upgraded to at least 30% [5]. Therefore, the development of technology for gas separation and purification is warranted. Usually, moisture, carbon dioxide, and other acidic gases can be removed in advance through simple and mature processes. However, the separation of methane and nitrogen is the most difficult and important step for the enrichment of low-concentration CBM, because the two components are similar in kinetic diameter (0.381 and 0.364 nm) and their critical temperatures are very low. 

Over the last few decades, CBM separation and purification technologies, including (mainly) solution absorption [6], cryogenic distillation [7], membrane separation [8], and pressure swing adsorption (PSA) [2], have been considered in numerous studies. Among them, cryogenic distillation is a relatively mature technology that is widely used; however, it is energy-intensive and costly [5,9] and usually requires high flow rates to sustain process economics [10]. PSA, as an alternative approach, is considered the most cost-effective method, because it needs fewer resources, including energy, and is highly efficient [9,10,11,12].

Adsorbents play an important role in the successful separation of methane and nitrogen using PSA technology [13]. Porous materials based on physisorption can probably be used for the separation and purification of CBM. Currently, the main adsorbents for CH_4_/N_2_ gas separation applications are activated charcoal, carbon molecular sieve, and zeolite molecular sieve [7,9,13]. In accordance with the reports related to the adsorptive property of methane–nitrogen binary gas mixtures [9,14,15,16], the desired absorbent is required to have high structural selectivity and high thermal stability. Among a wide range of porous materials, metal–organic frameworks (MOFs) have attracted particular interest, owing to their rich designability and high porosity [17,18,19].

MOF materials have traditionally been synthesized via solvothermal methods. Additionally, new MOF materials, with high CH_4_ capacity and excellent CH_4_/N_2_ adsorption selectivity, have been synthesized in recent years [20,21,22,23]. Zhou et al. reported a new flexible mesh-adjustable molecular sieve (MAMS-1), with a structure exhibiting the molecular door effect, i.e., the molecular door could open and close with various temperatures. At 113 K, the overall kinetic N_2_/CH_4_ selectivity was greater than 3 [20]. Senkovska et al. synthesized Cu_3_(btc)_2_ (HKUST-1) with a CH_4_ adsorption capacity of 16.5 wt.% at 298 K and 35 bar [21]. Similarly, Zhang et al. synthesized a novel Zn-based heterocycle metal–organic framework ZJU-197 (ZJU = Zhejiang University), which exhibited excellent CO_2_/CH_4_ and CO_2_/N_2_ adsorption selectivity [22]. Sumer et al. investigated the adsorption and diffusion of CH_4_/N_2_ mixtures in 102 different MOFs using molecular simulations and evaluated their adsorption-based and membrane-based separation performances. Three MOFs, BERGAI01, PEQHOK, and GUSLUC, exhibited the best adsorption selectivity [23].

The term “flexible MOFs” has been used to describe MOFs with stimuli-induced structural transformability of considerable amplitude [24]. Flexible MOF materials have become an important adsorbent (owing to their large specific surface area, adjustable pore size, and high porosity), however they are quite costly. 

Additionally, mesoporous SBA-15, as a representative mesoporous silica material, has attracted substantial attention in the fields of adsorption, separation, and catalysis [25,26,27]. Considering the background of CBM application, adsorbents with high performance/cost ratio are needed. The complex preparation process, low yield, and high price of MOFs would limit their application. Thus, by the comparison of several commercial materials, a synthetic spherical SBA-15 with large surface area and excellent adsorption performance is selected as the carrier of MOF structure. Ni, as a cheap metal with good affinity to methane, is chosen as the metal center of the MOF material in the present work. 

Therefore, an inexpensive adsorbent with excellent adsorption capacity and selectivity is needed for the enrichment of CH_4_/N_2_ gas mixture. In this study, two types of Ni-based MOF materials are synthesized, using a solvothermal method, on spherical SBA-15. The adsorption performance of the materials is compared, and the most suitable adsorbent is suggested for the adsorption and enrichment of CH_4_ from a CH_4_/N_2_ mixture.

## 2. Materials and Methods

### 2.1. Synthesis of Spherical SBA-15

In a typical synthesis, 3 g P123 was dissolved in a 60 mL solution of 1.5 mol/L HCl and vigorously stirred. Afterward, 0.6 g hexadecyl trimethyl ammonium bromide dissolved in 25 mL deionized water was added, prior to the addition of 20 mL absolute ethanol. The mixture was then heated to 35 °C, with 10 mL tetraethyl orthosilicate added in a dropwise manner. After stirring for 45 min, the mixture was heated statically at 75 °C under reflux for 5 h. Subsequently, the mixture was placed in a teflon-lined stainless steel autoclave and aged for 40 h at 80 °C. The mixture was then washed, dried, and calcined at 550 °C in air atmosphere, resulting in a white powder designated as SBA-15.

### 2.2. Synthesis of MOF-1/SBA-15

Firstly, 5-tert-butyl-1,3-benzenedicarboxylic acid (H_2_(bbdc), 375 mg) was dissolved in 7.5 mL ethylene glycol. Secondly, 750 mg nickel nitrate (Ni(NO_3_)_2_·6H_2_O) dissolved in 30 mL deionized water was added and the mixture was vigorously stirred [20]. A 500 mg SBA-15 sample was added during stirring and then placed in a teflon-lined stainless steel autoclave. The sample was statically heated at 210 °C for 24 h, and then slowly cooled to room temperature. The end product was then washed with a mixture of deionized water and methanol and dried under vacuum at room temperature, thereby yielding sample MOF-1/SBA-15. For comparison, pristine MOF-1 was prepared without the addition of SBA-15. 

### 2.3. Synthesis of MOF-2/SBA-15

*N*,*N*-Dimethylformamide (DMF, 40 mL) was added to a mixture of terephthalic acid (1,4-BDC, 332 mg, 2.0 mmol), isonicotinic acid (248 mg, 2.0 mmol), and nickel nitrate (Ni(NO_3_)_2_·6H_2_O, 1784 mg, 6.0 mmol) [28]. A 500 mg SBA-15 sample was added to the mixture, which was then vigorously stirred. The mixture was subsequently placed in a teflon-lined stainless steel autoclave, and after holding for 96 h at 85 °C, the sample was slowly cooled to room temperature. The end product was washed with DMF and dried under vacuum at room temperature, thereby yielding sample MOF-2/SBA-15. For comparison, pristine MOF-2 was prepared without the addition of SBA-15.

### 2.4. Characterization

The microstructure of the sample was observed via scanning electron microscopy (SEM; Hitachi S-4800, Tokyo, Japan) at a working voltage of 15.0 kV. The Ni and Si content was determined via X-ray fluorescence (XRF; ARL-9800 spectrometer, Beijing, China) elemental analysis of the samples. The content of organic C, H, N, and O was determined using the dynamic combustion method on an elemental analyzer (CHNS; Elementar, Vario Macro Cube, Langenselbold, Germany). The phase composition of the samples was evaluated by means of X-ray powder diffraction (XRD; Philips X’Pro diffractometer, Amsterdam, Netherlands) operating at a tube voltage and tube current of 40 kV and 40 mA, respectively, with Cu Kα radiation with a wavelength of 1.5418 Å. Scans were performed over 2θ of 0.8°–3.0° in the small-angle range and 5°–80° in the wide-angle range. Fourier-transform infrared spectroscopy (FT-IR) of the samples was conducted on a Bruker Vertex 80v spectrophotometer (Beijing, China) using KBr pellets. A total of 64 scans were recorded, with a resolution of 4 cm^−1^. The specific surface area and pore volume of each catalyst were obtained by means of N_2_ adsorption measurements (Micrometrics ASAP-2020, Atlanta, GE, USA) at 77 K. The samples were pretreated at 623 K under vacuum prior to N_2_ adsorption.

### 2.5. Adsorption Performance

High pressure adsorption isotherms were obtained from Micromeritics HPVA II (Mike, Atlanta, GE, USA). The sample was placed in an oven for 3 h at 150 °C and then degassed for 16 h at 200 °C under vacuum (pressure < 0.03 bar). The sample tube was then cooled to room temperature and placed in a constant temperature water bath (Julabo F25-HE, accuracy: 0.01 °C). Afterward, the pressure was evacuated to and maintained at 0.02 bar for 30 min prior to the test. The test pressure and pressure balance condition were set such that the pressure changes by < 0.003 bar within 3 min. After testing free space, the adsorption–desorption curve of the sample in gas was determined. A high purity CH_4_ (≥ 99.999%) and N_2_ (≥ 99.999%) adsorption gas was employed. 

Low pressure adsorption measurements at pressure ranging from 50 to 150 kPa were performed on a self-built micro-reactor equipped with a thermal conductivity detector (TCD, Jingke, Shanghai, China). A total of 200 mg of the sample (0.25~0.35 mm diameter) was placed in a fixed-bed straight stainless steel pipe reactor. The temperature of the reactor was monitored by a K-type thermocouple and connected to a temperature-indicator controller. The sample was pretreated at 150 °C in helium (He) flow for 2 h, cooled to room temperature, and then vacuumed to 8 kPa. After the test pressure and temperature were set and remained static, the adsorption gas diluted with He was flowed past the sample bed, and the change of gas content was detected by TCD. Corresponding adsorption and desorption amounts were calculated from the TCD results. The Langmuir model was used to determine the correlation between the adsorption selectivity of CH_4_ and N_2_ on the samples. A high purity He (≥ 99.999%), CH_4_ (≥ 99.999%), and N_2_ (≥ 99.999%) adsorption gas was employed. 

## 3. Results and Discussion

### 3.1. Scanning electron microscopy (SEM) Images

**Figure 1:**
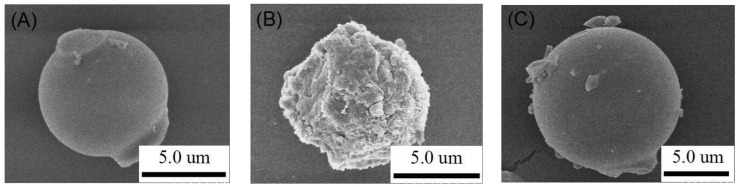
SEM images of the samples: (**A**) spherical SBA-15; (**B**) MOF-1/SBA-15; (**C**) MOF-2/SBA-15.

As revealed via SEM images (see Figure 1), the prepared samples are all uniformly spherical with sizes ranging from 5 to 10 μm. SBA-15 has a distinct spherical structure with a relatively smooth surface (Figure 1A). MOF-2/SBA-15 also has a spherical structure (albeit with a few attachments on the surface; see Figure 1C). This suggests that spherical SBA-15 plays an important role in the morphological characteristics of MOF-2/SBA-15. However, as shown in Figure 1B, the MOF-1/SBA-15 sample appears as an accumulation of massive structures with a rough surface, indicating that the structure of spherical SBA-15 might have been partially destroyed during the synthesis process.

### 3.2. X-ray fluorescence (XRF) and Elemental Analysis

The composition of the samples (see Table 1) was determined via XRF and CHNS analysis, with inorganic and organic components tested, respectively. SiO_2_ is the main component of SBA-15, while NiO constitutes 14.0 ± 0.02 wt % and 13.0 ± 0.01 wt % of MOF-1/SBA-15 and MOF-2/SBA-15, respectively, consistent with the amount of reagents added during the synthesis process. Therefore, Ni has been well integrated with SBA-15. Additionally, from the result of CHNS analysis, the contents of C, H, N, and O elements are basically consistent with the organic composition in the raw materials, with solvents included. The total organic contents of MOF-1/SBA-15 and MOF-2/SBA-15 are 38.2 ± 0.035% and 43.7 ± 0.057% by weight, respectively. Therefore, the samples should be activated for the removal of solvents before the test of the adsorption performance.

### 3.3. X-ray powder diffraction (XRD) Patterns

**Figure 2:**
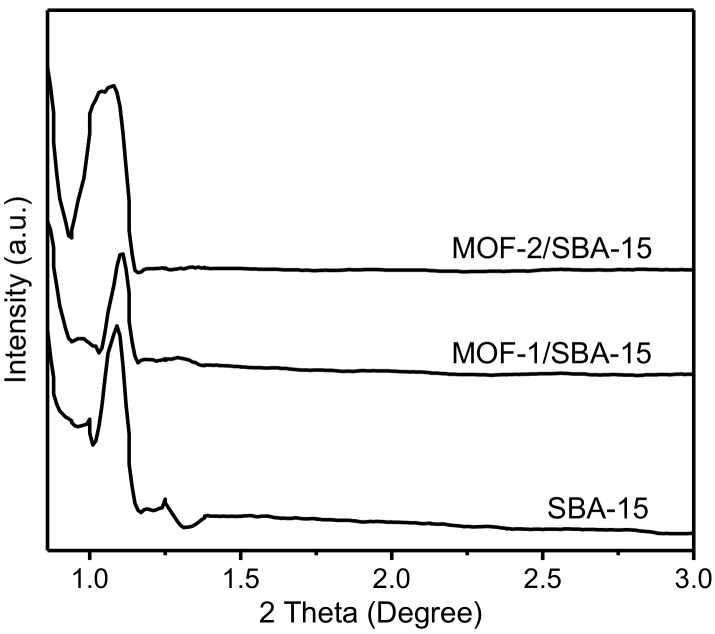
Small-angle X-ray diffraction (SXRD) patterns of spherical SBA-15, MOF-1/SBA-15 and MOF-2/SBA-15.

Small–angle X-ray diffraction (SXRD) can accurately characterize the organization of materials and is one of the main characterization techniques used for the identification of ordered mesoporous structures. The SXRD patterns of the samples are shown in Figure 2. The characteristic pattern of SBA-15, with a high-intensity reflection at 2θ around 1.1° (corresponding to the (100) plane that can be indexed to a cavity with hexagonal symmetry), is observed for each material [29,30,31,32]. The results in Figure 2 indicate that the ordered mesoporous structure has been retained after the modification with MOF-1 or MOF-2, although the morphology of MOF-1/SBA-15 differs from that of MOF-2/SBA-15. 

**Figure 3:**
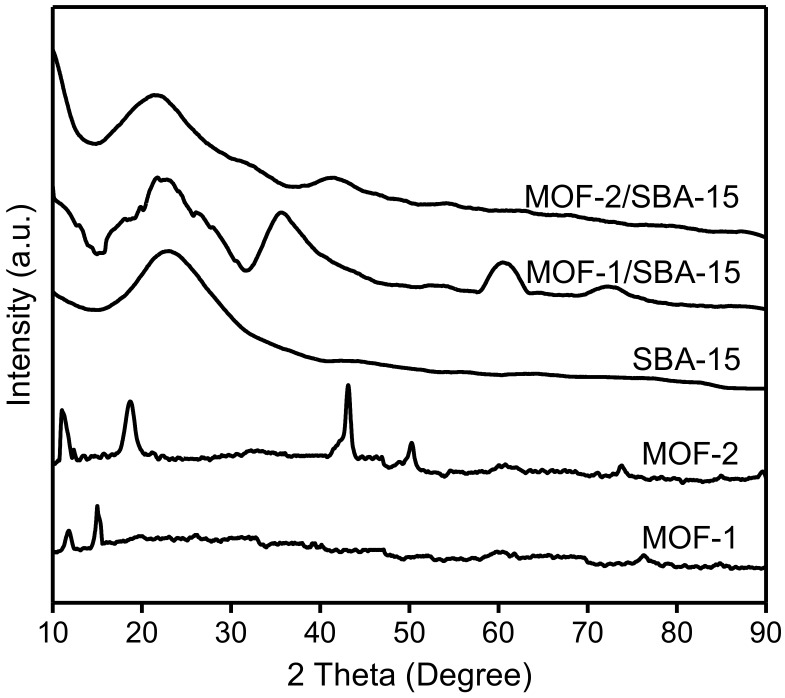
Wide-angle X-ray diffraction (WXRD) patterns of spherical SBA-15, MOF-1, MOF-2, MOF-1/SBA-15 and MOF-2/SBA-15.

The wide–angle X-ray diffraction (WXRD) patterns of the samples are shown in Figure 3. For spherical SBA-15, the peak occurring at 2θ = 22.7° coincides exactly with that reported on the Joint Committee on Powder Diffraction Standards (JCPDS) card, indicating that SBA-15 has been successfully synthesized [29]. WXRD patterns were obtained to elucidate the crystal structures of pristine MOF-1 and MOF-2. The strong diffraction peaks (at 11.8° and 15.1° for MOF-1; at 11.1°, 18.7°, 42.9°, and 50.3° for MOF-2) indicate certain crystallinity of both samples and mean the formation of micropores [33]. For MOF-1/SBA-15, peaks occur at 2θ = 35.7°, 60.8°, and 72.6°, corresponding to the (111), (220), and (311) reflections of NiO, respectively, which indicates the partial decomposition of MOF-1 during the formation of the composite material [34]. The main diffraction peaks of MOF-2/SBA-15 appear with relatively low intensity at 2θ = 20.8°, 32.3°, 41.4°, and 53.9°, indicating that the incorporation of MOF-2 into SBA-15 is accompanied by blockage of some pores, with the existence of some Ni metals [35]. Therefore, MOF-1/SBA-15 and MOF-2/SBA-15 inherited the parent structure of SBA-15, even in the presence of Ni species.

### 3.4. Fourier-transform infrared spectroscopy (FT-IR)

**Figure 4:**
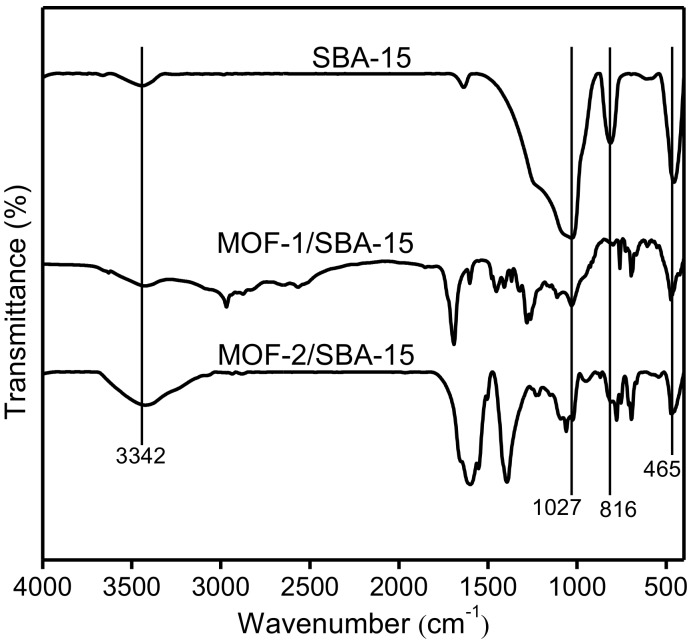
FT-IR spectra of spherical SBA-15, MOF-1/SBA-15 and MOF-2/SBA-15.

Figure 4 shows the FT-IR spectra of spherical SBA-15, MOF-1/SBA-15, and MOF-2/SBA-15. Each spectrum is characterized by absorption peaks at 1027, 816, and 465 cm^−1^, arising from the asymmetric and symmetric stretching vibrations, and bending modes, respectively, of the Si–O–Si framework. The broad band appeared at 3432 cm^−1^ is attributed to O–H stretching [36]. For MOF-1/SBA-15 and MOF-2/SBA-15, the absorption peak at 698 cm^−1^ is attributed to the vibration mode of O–Ni–O [37]. Meanwhile, several other peaks appear at wavenumbers ranging from 400 to 1800 cm^−1^, indicating the occurrence other functional groups. The bands at around 756 and 1058 cm^−1^ are attributed to the vibrational modes of phenyl ring, and the asymmetric and symmetric vibration of –COOH are observed at 1388 and 1604 cm^−1^ [38]. A strong band appears at 1275 cm^−1^ for MOF-1/SBA-15, due to the stretching vibration of N–C, indicating the existence of DMF.

### 3.5. Nitrogen Adsorption–Desorption

**Figure 5:**
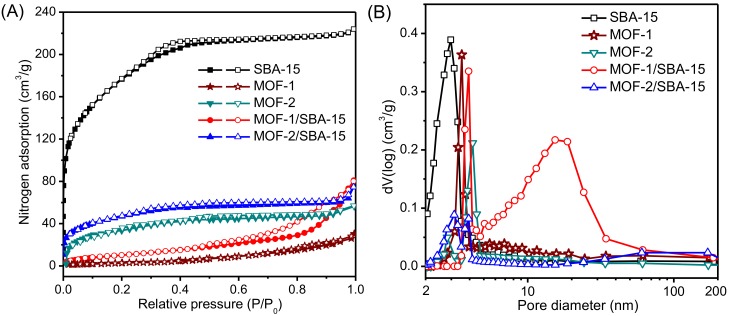
(**A**) Nitrogen adsorption–desorption isotherms of the samples; (**B**) BJH pore size distribution of the samples.

The porosity and textural property of the samples are determined from nitrogen adsorption–desorption isotherms (see Figure 5 for isotherms and corresponding Barrett-Joyner-Halenda (BJH) pore size distributions of the samples). The BET surface area and total pore volume are listed in Table 2. Meanwhile, pristine MOF-1 and MOF-2 were characterized for comparison.

According to International Union of Pure Chemical and Applied Chemistry (IUPAC), the nitrogen adsorption–desorption isotherms of SBA-15, MOF-2, and MOF-2/SBA-15 can be characterized as type I [34]. For *P*/*P*_0_ at around 0.0, the isotherm shows a sharp increase attributed to the micropores. The blend type IV is observed in MOF-1 and MOF-1/SBA-15 [5,39]. The isotherm corresponding to SBA-15 exhibits an obvious inflection at *P*/*P*_0_ ranging from 0.3 to 0.6. This inflection results from the capillary condensation of nitrogen within pores with an H_2_-type hysteresis loop [40,41]. The isotherms of two MOF/SBA-15 samples are similar to their pristine MOF structures, but have higher adsorption capacity. The hysteresis loop of MOF-1/SBA-15 is shifted to higher *P*/*P*_0_ (from 0.4 to 1.0) relative to that of SBA-15, and shows evidence of slit hole structures due to the aggregation of MOFs. For the MOF-2/SBA-15 sample, the *P*/*P*_0_ range of the hysteresis loop is narrower (from 0.3 to 0.6) than that of MOF-1/SBA-15, indicative of a different pore distribution. 

The BJH pore size distributions are compared (Figure 5B). The distribution of SBA-15 is uniform and narrow with an average pore diameter of 3.0 ± 0.15 nm. Average pore sizes of 3.6 ± 0.08 nm and 4.2 ± 0.21 nm are obtained for MOF-1 and MOF-2, respectively. Many 15-nm-diameter pores occur in the MOF-1/SBA-15 sample, possibly owing to accumulation of the structures (see the SEM images in Figure 1B). Compared with that of MOF-1/SBA-15, the pore size distribution of MOF-2/SBA-15 is more uniform, indicating that MOF-2/SBA-15 exhibits better CH_4_/N_2_ adsorption selectivity than MOF-1/SBA-15. 

A high specific surface area (i.e., 638 ± 9.6 m^2^/g; see Table 2) is determined for sample SBA-15. However, this area decreases when MOF-1 or MOF-2 is synthesized on SBA-15. During the synthesis process, the pores of SBA-15 might be damaged, resulting in the decrease of surface area [42,43]. This is especially true for MOF-1, as evidenced by a surface area of 43 m^2^/g for the MOF-1/SBA-15 sample. The two pristine materials, MOF-1 and MOF-2, mainly exhibit a flocculent structure, but are extremely easy to agglomerate, with specific surface areas of only 26 ± 1.3 and 57 ± 2.1 m^2^/g, respectively. The existence of SBA-15 could facilitate the dispersion of MOF material. Similarly, consistent with the pore size distribution result, the total pore volume of MOF-1/SBA-15 and MOF-2/SBA-15 are both significantly lower than that of SBA-15, but slightly higher than the pristine materials.

The adsorption capacity of materials is affected by the BET surface area and pore size distribution, which also determine the capacity of samples for the selective adsorption of CH_4_ [44].

### 3.6. High Pressure Adsorption Isotherm

For a given temperature and various pressures, adsorption isotherms can describe the equilibrium gas-adsorption capacity of adsorbents. The shape of the adsorption isotherm varies with the pore structure of the material. According to the IUPAC classification, there are six different types of adsorption isotherms, however only type I, II, IV, and VI curves are applicable to porous materials [45]. 

**Figure 6:**
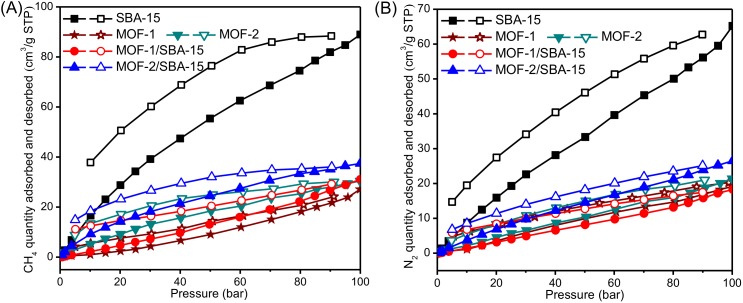
Adsorption and desorption equilibrium isotherms of CH_4_ (**A**) and N_2_ (**B**) for the samples.

The adsorption isotherms of CH_4_ and N_2_ at 25 ^°^C are measured for the SBA-15, MOF-1, MOF-2, MOF-1/SBA-15, and MOF-2/SBA-15 samples (see Figure 6 for the corresponding results). Their adsorption curves exhibit characteristics (e.g., hysteresis in the isotherms) consistent with those of a type IV curve. Adsorption and desorption processes are only partly reversible and, therefore, their isotherms differ. Although the adsorption and desorption isotherms are not closed completely, they basically follow the same path, suggesting that the adsorbed molecules can be recovered and the adsorbents can be regenerated during the desorption process by decreasing the pressure to atmospheric pressure or vacuum condition, if necessary. As shown in Figure 6, the adsorption amount and the retaining loop area of spherical SBA-15 are significantly larger than those of MOF-1, MOF-2, MOF-1/SBA-15 and MOF-2/SBA-15. This indicates that, of the samples, spherical SBA-15 has the best CH_4_ and N_2_ adsorption capacity at elevated pressures, suggesting that it is the best adsorbents for CH_4_ or N_2_ storage. The MOF structures may have occupied some of the pores in SBA-15, resulting in fewer adsorption amounts than that of the SBA-15 sample. MOF-1/SBA-15 and MOF-2/SBA-15 show higher adsorption amounts than pristine MOF-1 and MOF-2, benefitted from the mesoporous structures with the participation of SBA-15.

### 3.7. Determination of Adsorption Selectivity Parameters

In order to evaluate the adsorption selectivity and predict the adsorption performance of gas mixture from pure component isotherms, low pressure adsorption of CH_4_ and N_2_ was measured at 25 ^°^C, with the pressure ranging from 50 to 150 kPa, i.e., *P*/*P*_0_ = 0.5~1.5, as listed in Table 3. This pressure range represents the typical vacuum pressure swing adsorption conditions for CBM enrichment.

**Figure 7:**
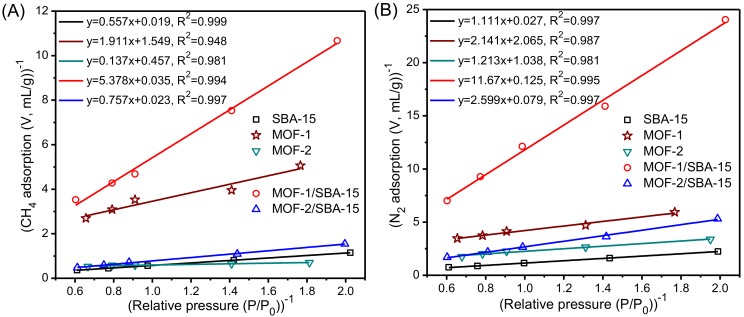
(1/*V*) versus (1/*P*) plots of CH_4_ (**A**) and N_2_ (**B**) for the samples. Symbols: experimental values; ―― linear fitting to the Langmuir equation.

The Langmuir model is used to fit the isotherms and determine the correlation between CH_4_ and N_2_ adsorption on the samples [44]. The Langmuir adsorption Equation (1) can be transformed to Equation (2):

(1) $$V=\frac{V_{m}BP}{1+BP}$$

(2) $$\frac{1}{V}=\frac{1}{BV_{m}}\cdot \frac{1}{P}+\frac{1}{V_{m}}$$

where *V* is the volume of the adsorbed gas in standard state when the partial gas pressure is *P*. The Langmuir isotherm equation parameters *V*_m_ (mL/g) and *B* can be determined from the slope and intercept of a linear Langmuir plot of (1/*V*) versus (1/*P*), see equation (2) [46,47]. The adsorption values of the samples in Table 3 were processed and plotted, respectively, and Langmuir equation was used to fit the points with the fitted equation and correlation coefficient R^2^ provided (Figure 7). 

Knowledge of the adsorption capacity and selectivity of the adsorbent is essential for evaluating the efficiency of the adsorbent for the adsorption-induced enrichment of CH_4_ from the gas mixture. The adsorption equilibrium selectivity of a CH_4_/N_2_ gas mixture is defined as:

(3) $$\alpha_{CH_{4}/N_{2}}=\frac{X_{CH_{4}}}{X_{N_{2}}}\cdot \frac{Y_{N_{2}}}{Y_{CH_{4}}}=\frac{b_{N_{2}}}{b_{CH_{4}}}$$

where *X*_CH__4_ and *X*_N__2_ are the molar fractions of CH_4_ and N_2_ in the adsorbed phase, *Y*_CH__4_ and *Y*_N__2_ are the molar fractions of CH_4_ and N_2_ in the gas phase, *b*_N2_ is the slope of the N_2_ line and *b*_CH4_ is the slope of the CH_4_ line. Thus, the α_CH__4/N__2_ values of the samples can be calculated from the results of Figure 7. Table 4 lists the adsorption selectivity parameters of the samples, with some reported results included [46,47].

In the enrichment and separation process, the adsorption amount will determine the concentration of gas released. Therefore, the value of the saturated gas adsorption (*V*_CH__4_ and *V*_N__2_) at 150 kPa represents an important parameter in selecting the adsorbent. 

The adsorption capacity ratio of two components under varying pressures, i.e., the adsorption capacity selection coefficient, *W*, plays an important role in the adsorption separation. This capacity refers mainly to the adsorption amount of a component under high and low adsorption pressures, and can be calculated from the adsorption isotherm of the pure components. For example, consider the case of CH_4_ and N_2_:

(4) $$W_{CH_{4}/N_{2}}=\frac{\Delta V_{CH_{4}}}{\Delta V_{N_{2}}}=\frac{V_{CH_{4}(150\mathrm{kPa})}-V_{CH_{4}(50\mathrm{kPa})}}{V_{N_{2}(150\mathrm{kPa})}-V_{N_{2}(50\mathrm{kPa})}}$$

where *ΔV*_CH__4_ and *ΔV*_N__2_ are, for CH_4_ and N_2_, respectively: working capacity calculated as the adsorption equilibrium capacity difference at an adsorption pressure of 150 kPa and a desorption pressure of 50 kPa (see Table 4).

For pressure swing adsorption process, the adsorbent selection parameter *S* is more useful in adsorbent evaluation and selection [44,46], which can then be determined from:

(5) $$S_{CH_{4}/N_{2}}=\alpha_{CH_{4}/N_{2}}\cdot W_{CH_{4}/N_{2}}$$

The adsorbent selection parameter *S* can be used to compare the adsorption performance of various absorbents. The value of *S* increases with the improving adsorption performance of the adsorbent.

From Table 4, the samples may be written in descending order of the adsorption equilibrium selectivity α_CH__4/N__2_ and adsorbent selection parameter *S*_CH__4/N__2_ as follows: MOF-2/SBA-15 > MOF-2 > MOF-1/SBA-15 > SBA-15 > MOF-1. MOF-1/SBA-15 and MOF-2/SBA-15 show higher adsorption selectivity than pristine MOF-1 and MOF-2, indicating that the composition of MOF structure and SBA-15 facilitates the selectivity for CH_4_ adsorption. MOF-2/SBA-15 exhibits the best adsorption selectivity, as evidenced by its *α*_CH__4/N__2_ value of 3.44 and *S*_CH__4/N__2_ value of 11.1. For equilibrium adsorption, the *α*_CH__4/N__2_ value of MOF-2/SBA-15 is slightly lower than the reported MOF-177 and activated carbon; for pressure swing adsorption, the *S*_CH__4/N__2_ value of MOF-2/SBA-15 is higher than the reported results in the same conditions [46,47]. Anyway, the adsorption capacity of spherical SBA-15 is the largest among the samples. Therefore, MOF-2/SBA-15 should be further improved to meet the requirement of the most suitable adsorbent for the adsorption enrichment of the CH_4_/N_2_ mixture.

## 4. Conclusions

In summary, two types of Ni-based MOF/SBA-15 composite materials, MOF-1/SBA-15 and MOF-2/SBA-15, have been successfully synthesized and characterized. The results of the structural characterization and adsorption performance test revealed that the MOF-2/SBA-15 sample has the best CH_4_/N_2_ adsorption selectivity, with an adsorbent selection parameter (*S*) of 11.1. Spherical SBA-15 has the largest BET surface area, pore volume, and (in turn) adsorption capacity. This may have resulted from the fact that some of the mesopores of SBA-15 were plugged during the synthesis of MOF-2/SBA-15. Further research and optimization of spherical SBA-15-based MOF materials are required for achieving both excellent adsorption selectivity and large adsorption capacity. These materials may provide new methods for the effective enrichment of CH_4_ from the CH_4_/N_2_ mixture.

## Figures and Tables

**Table 1:** The composition of the samples.

Samples	Inorganic Component (wt %)		Organic Component (wt %)			
	SiO_2_	NiO	C	H	N	O
SBA-15	100	-	-	-	-	-
MOF-1/SBA-15	47.8 ± 0.07	14.0 ±0.02	21.5 ± 0.02	2.8 ± 0.005	-	13.9 ± 0.01
MOF-2/SBA-15	43.3 ±0.06	13.0 ±0.01	23.5 ± 0.03	2.8 ± 0.004	4.0 ± 0.003	13.4 ± 0.02

**Table 2:** Textural properties of the samples from nitrogen adsorption–desorption results.

Samples	BET Surface Area (m^2^/g)	Average Pore Diameter (nm)	Total Pore Volume (cm^3^/g)
SBA-15	638 ± 9.6	3.0 ± 0.15	1.28 ± 0.09
MOF-1	26 ± 1.3	3.6 ± 0.08	0.25 ± 0.04
MOF-2	57 ± 2.1	4.2 ± 0.21	0.31 ± 0.03
MOF-1/SBA-15	43 ± 2.3	4.0 ± 0.33	0.53 ± 0.04
MOF-2/SBA-15	171 ± 3.5	3.1 ± 0.17	0.43 ± 0.02

**Table 3:** The low-pressure adsorption capacity of CH_4_ and N_2_ on the samples.

Samples	CH_4_ Adsorption		N_2_ Adsorption	
	*P*/*P*_0_	*V*_CH__4_ (mL/g)	*P*/*P*_0_	*V*_N__2_ (mL/g)
SBA-15	0.49	0.87	0.50	0.45
	0.70	1.24	0.70	0.62
	1.02	1.78	1.00	0.88
	1.29	2.24	1.32	1.15
	1.64	2.72	1.64	1.34
MOF-1	0.57	0.20	0.56	0.17
	0.71	0.26	0.76	0.20
	1.08	0.28	1.10	0.24
	1.25	0.32	1.27	0.27
	1.52	0.37	1.53	0.29
MOF-2	0.55	1.42	0.51	0.30
	0.71	1.54	0.76	0.38
	1.10	1.68	1.12	0.45
	1.26	1.72	1.28	0.50
	1.52	1.89	1.47	0.57
MOF-1/SBA-15	0.51	0.09	0.49	0.04
	0.72	0.14	0.71	0.06
	1.11	0.22	1.01	0.08
	1.27	0.23	1.29	0.11
	1.66	0.28	1.66	0.14
MOF-2/SBA-15	0.50	0.65	0.50	0.19
	0.69	0.92	0.71	0.27
	1.14	1.39	1.02	0.38
	1.33	1.69	1.23	0.46
	1.63	2.11	1.66	0.59

**Table 4:** Summary of the parameters related to the adsorption selectivity of the samples calculated from the pure gas adsorption performances.

Samples	Adsorption Equilibrium Amount (mL/g)			
	At an Adsorption Pressure of 150 kPa		At a Desorption Pressure of 50 kPa	
	*V*_CH__4_	*V*_N__2_	*V*_CH__4_	*V*_N__2_
SBA-15	2.56	1.30	1.23	0.58
MOF-1	0.35	0.29	0.21	0.18
MOF-2	1.82	0.54	0.49	0.41
MOF-1/SBA-15	0.28	0.13	0.15	0.07
MOF-2/SBA-15	1.89	0.55	0.90	0.24
	**Parameters related to the adsorption selectivity**			
	***α*** **_CH_** **_4/N_** **_2_**	***W*** **_CH_** **_4/N_** **_2_**	***S*** **_CH_** **_4/N_** **_2_**	**Reference**
SBA-15	1.99	1.84	3.66	
MOF-1	1.12	1.39	1.56	
MOF-2	2.66	1.93	5.13	
MOF-1/SBA-15	2.17	2.19	4.75	
MOF-2/SBA-15	3.44	3.24	11.1	
MOF-5	1.13	-	0.67	[46]
MOF-177	4.00	-	8.45	[46]
Zeolite 5A	0.94	-	0.81	[46]
Activated carbon	4.60	-	4.02	[47]

## References

[1] Simon C.M., Kim J., Gomez-Gualdron D.A., Camp J.S., Chung Y.G., Martin R.L., Mercado R., Deem M.W., Gunter D., Haranczyk M. (2015). The materials genome in action: Identifying the performance limits for methane storage. Energy Environ. Sci..

[2] Kim S., Ko D., Row S., Kim J. (2016). Techno-economic evaluation of hybrid systems of pressure swing adsorption and membrane processes for coalbed methane separation. Chem. Eng. Res. Des..

[3] Zou W.F. (2016). Proceedings of the 2016 6th International Conference on Applied Science, Engineering and Technology (ICASET). Adv. Eng. Res..

[4] Karakurt I., Aydin G., Aydiner K. (2011). Mine ventilation air methane as a sustainable energy source. Renew. Sustain. Energy Rev..

[5] Hao X.F., Hu H.J., Li Z., Wu L.M., Liu X.Q., Zhang Y.N. (2018). Adsorption properties of modified clinoptilolite for methane and nitrogen. Materials.

[6] Mehra Y.R. (1986). Utilizing the Mehra Process for Processing and BTU Upgrading of Nitrogen-Rich Natural Gas Streams. US Patent.

[7] Li P.Y., Tezel F.H. (2007). Adsorption separation of N_2_, O_2_, CO_2_ and CH_4_ gases by beta-zeolite. Microporous Mesoporous Mater..

[8] Huang Y., Paul D.R. (2007). Effect of film thickness on the gas-permeation characteristics of glassy polymer membranes. Ind. Eng. Chem. Res..

[9] Mulgundmath V.P., Tezel F.H., Hou F., Golden T.C. (2012). Binary adsorption behaviour of methane and nitrogen gases. J. Porous Mater..

[10] Cavenati S., Grande C.A., Rodrigues A.E. (2006). Separation of CH_4_/CO_2_/N_2_ mixtures by layered pressure swing adsorption for upgrade of natural gas. Chem. Eng. Sci..

[11] Liu Y.L., Liu Y.S., Yang X. (2013). Proportion pressure swing adsorption for low concentration coal mine methane enrichment. Sep. Sci. Technol..

[12] Yi H.H., Li F.R., Ning P., Tang X.L., Peng J.H., Li Y.D., Deng H. (2013). Adsorption separation of CO_2_, CH_4_, and N_2_ on microwave activated carbon. Chem. Eng. J..

[13] Cavenati S., Grande C.A., Rodrigues A.E. (2005). Separation of methane and nitrogen by adsorption on carbon molecular sieve. Sep. Sci. Technol..

[14] Liu B., Smit B. (2009). Comparative molecular simulation study of CO_2_/N_2_ and CH_4_/N_2_ separation in zeolites and metal-organic frameworks. Langmuir.

[15] Rufford T.E., Watson G.C.Y., Saleman T.L., Hofman P.S., Jensen N.K., May E.F. (2013). Adsorption equilibria and kinetics of methane plus nitrogen mixtures on the activated carbon norit RB3. Ind. Eng. Chem. Res..

[16] Saleman T.L.H., Watson G.C.Y., Rufford T.E., Hofman P.S., Chan K.I., May E.F. (2013). Capacity and kinetic measurements of methane and nitrogen adsorption on H^+^-mordenite at 243–303 K and pressures to 900 kPa using a dynamic column breakthrough apparatus. Adsorption.

[17] Lu W.G., Wei Z.W., Gu Z.Y., Liu T.F., Park J., Park J., Tian J., Zhang M.W., Zhang Q., Gentle T. (2014). Tuning the structure and function of metal-organic frameworks via linker design. Chem. Soc. Rev..

[18] Bai Y., Dou Y.B., Xie L.H., Rutledge W., Li J.R., Zhou H.C. (2016). Zr-based metal-organic frameworks: Design, synthesis, structure, and applications. Chem. Soc. Rev..

[19] Furukawa S., Reboul J., Diring S., Sumida K., Kitagawa S. (2014). Structuring of metal-organic frameworks at the mesoscopic/macroscopic scale. Chem. Soc. Rev..

[20] Ma S.Q., Sun D.F., Wang X.S., Zhou H.C. (2007). A mesh-adjustable molecular sieve for general use in gas separation. Angew. Chem. Int. Edit..

[21] Senkovska I., Kaskel S. (2008). High pressure methane adsorption in the metal-organic frameworks Cu_3_(btc)_2_, Zn_2_(bdc)_2_dabco, and Cr_3_F(H_2_O)_2_O(bdc)_3_. Microporous Mesoporous Mater..

[22] Zhang L., Jiang K., Yang Y., Cui Y.J., Chen B.L., Qian G.D. (2017). A novel Zn-based heterocycle metal-organic framework for high C_2_H_2_/C_2_H_4_, CO_2_/CH_4_ and CO_2_/N_2_ separations. J. Solid State Chem..

[23] Sumer Z., Keskin S. (2017). Adsorption- and membrane-based CH_4_/N_2_ separation performances of MOFs. Ind. Eng. Chem. Res..

[24] Chang Z., Yang D.H., Xu J., Hu T.L., Bu X.H. (2015). Flexible metal-organic frameworks: Recent advances and potential applications. Adv. Mater..

[25] Sayari A., Han B.H., Yang Y. (2004). Simple synthesis route to monodispersed SBA-15 silica rods. J. Am. Chem. Soc..

[26] Wan H.H., Liu L., Li C.M., Xue X.Y., Liang X.M. (2009). Facile synthesis of mesoporous SBA-15 silica spheres and its application for high-performance liquid chromatography. J. Colloid Interface Sci..

[27] Song S.W., Hidajat K., Kawi S. (2005). Functionalized SBA-15 materials as carriers for controlled drug delivery: Influence of surface properties on matrix-drug interactions. Langmuir.

[28] Shi F.J. (2012). Synthesis, Characterization and Gas Adsorption Behaviors of Meso- and Microporous Molecular Sieves and Metal-Organic Framework Materials. Ph.D. Thesis.

[29] Usami Y., Hongo T., Yamazaki A. (2012). Thermal stability and behavior of platelet-shaped SBA-15 containing Zr. J. Porous Mater..

[30] Wang J.H., Ge H.G., Bao W.R. (2015). Synthesis and characteristics of SBA-15 with thick pore wall and high hydrothermal stability. Mater. Lett..

[31] Yang H., Vovk G., Coombs N., Sokolov I., Ozin G.A. (1998). Synthesis of mesoporous silica spheres under quiescent aqueous acidic conditions. J. Mater. Chem..

[32] Martins A.R., Cunha I.T., Oliveira A.A.S., Moura F.C.C. (2017). Highly ordered spherical SBA-15 catalysts for the removal of contaminants from the oil industry. Chem. Eng. J..

[33] Sherino B., Mohamad S., Halim S.N.A., Manan N.S.A. (2018). Electrochemical detection of hydrogen peroxide on a new microporous Ni–metal organic framework material-carbon paste electrode. Sens. Actuators B Chem..

[34] Nakatsuka K., Yoshii T., Kuwahara Y., Mori K., Yamashita H. (2018). Controlled pyrolysis of Ni-MOF-74 as a promising precursor for the creation of highly active Ni nanocatalysts in size-selective hydrogenation. Chem. Eur. J..

[35] Kim J.H., Kang S.H., Zhu K., Kim J.Y., Neale N.R., Frank A.J. (2011). Ni-NiO core-shell inverse opal electrodes for supercapacitors. Chem. Commun..

[36] Arul P., John S.A. (2018). Size controlled synthesis of Ni-MOF using polyvinylpyrrolidone: New electrode material for the trace level determination of nitrobenzene. J. Electroanal. Chem..

[37] Guo H., Zheng Z., Zhang Y., Lin H., Xu Q. (2017). Highly selective detection of Pb^2+^ by a nanoscale Ni-based metal-organic framework fabricated through one-pot hydrothermal reaction. Sens. Actuators B Chem..

[38] Liang R., Shen L., Jing F., Wu W., Qin N., Lin R., Wu L. (2015). NH_2_-mediated indium metal-organic framework as a novel visible-light-driven photocatalyst for reduction of the aqueous Cr(VI). Appl. Catal. B Environ..

[39] Rexer T.F.T., Benham M.J., Aplin A.C., Thomas K.M. (2013). Methane adsorption on shale under simulated geological temperature and pressure conditions. Energy Fuel.

[40] Sing K.S.W., Everett D.H., Haul R.A.W., Moscou L., Pierotti R.A., Rouquerol J., Siemieniewska T. (1985). Reporting physisorption data for gas/solid systems—With special reference to the determination of surface area and porosity. Pure Appl. Chem..

[41] Meléndez-Ortiz H.I., Puente-Urbina B., Leona G.C., Mata-Padilla J.M., García-Uriostegui L. (2016). Synthesis of spherical SBA-15 mesoporous silica. Influence of reaction conditions on the structural order and stability. Ceram. Int..

[42] Pang J.B., Qiu K.Y., Wei Y. (2001). A new nonsurfactant pathway to mesoporous silica materials based on tartaric acid in conjunction with metallic chloride. Chem. Mater..

[43] Zheng J.Y., Pang J.B., Qiu K.Y., Wei Y. (2001). Synthesis and characterization of mesoporous titania and silica-titania materials by urea templated sol-gel reactions. Microporous Mesoporous Mater..

[44] Yang R.T. (2003). Adsorbents: Fundamentals and Applications.

[45] Wu H., Simmons J.M., Liu Y., Brown C.M., Wang X.S., Ma S. (2010). Metal-organic frameworks with exceptionally high methane uptake: Where and how is methane stored?. Chem. Eur. J..

[46] Saha D., Bao Z.B., Jia F., Deng S.G. (2010). Adsorption of CO_2_, CH_4_, N_2_O, and N_2_ on MOF-5, MOF-177, and Zeolite 5A. Environ. Sci. Technol..

[47] Liu Y.S., Guo G.D., Yang X., Li Y.L., Zhang H., Meng Y. (2010). Selection experiment of adsorbent for coal-bed methane enrichment by pressure swing adsorption process. Min. Saf. Environ. Prot..

